# Obstructive Pyelonephritis Secondary to Ureteral Compression by an Intra‐Abdominal Desmoid Tumor in Familial Adenomatous Polyposis: A Case Report

**DOI:** 10.1002/ccr3.73328

**Published:** 2026-08-09

**Authors:** Alireza Mehrban, Mehdi Karimi, Mohammad Taghi Hamidian, Mahdi Masoumi, Elahe Tari Najarkolaei, Abdolreza Babamahmoodi

**Affiliations:** ^1^ Clinical Department, Amol Campus of Medicine Mazandaran University of Medical Sciences (MazUMS) Sari Iran; ^2^ Faculty of Medicine Bogomolets National Medical University (NMU) Kyiv Ukraine; ^3^ Babol University of Medical Sciences Babol Iran; ^4^ Department of Radiology, School of Medicine Tehran University of Medical Sciences Tehran Iran; ^5^ Antimicrobial Resistance Research Center, Communicable Diseases Institute Mazandaran University of Medical Sciences Sari Iran

**Keywords:** case report, desmoid tumor, familial adenomatous polyposis, hydronephrosis, obstructive pyelonephritis, ureteral obstruction

## Abstract

In patients with familial adenomatous polyposis, intra‐abdominal desmoid tumors should be recognized as a rare but important cause of ureteral obstruction and obstructive pyelonephritis. Prompt cross‐sectional imaging, early recognition, and multidisciplinary management are essential for preserving renal function, treating infection, and preventing irreversible urinary tract complications.

## 1 Introduction

Familial adenomatous polyposis (FAP) is an autosomal dominant hereditary cancer syndrome caused by pathogenic variants in the APC gene. In addition to the development of numerous colorectal adenomas, patients may develop extracolonic manifestations, including desmoid tumors, osteomas, epidermoid cysts, and other benign lesions [1]. Desmoid tumors are among the most clinically significant extracolonic manifestations because of their locally aggressive behavior and potential to compress adjacent structures despite lacking metastatic potential [2].

Among these manifestations, desmoid tumors pose a particularly challenging management problem. These histologically benign but locally aggressive fibroblastic neoplasms frequently arise intra‐abdominally, especially in the mesentery or abdominal wall, following surgery in FAP patients. Their locally infiltrative growth pattern may result in encasement or compression of adjacent structures, potentially leading to clinically significant complications [3]. Although rare, ureteral involvement by intra‐abdominal desmoids is a recognized, serious complication that can cause obstructive uropathy, manifesting as hydronephrosis and potentially leading to obstructive pyelonephritis and renal impairment [4, 5, 6]. While documented in case reports, the diagnosis of this specific complication can be elusive due to nonspecific symptoms and the need for advanced cross‐sectional imaging, such as computed tomography (CT) or magnetic resonance imaging (MRI), for definitive characterization.

This study reports a rare clinically instructive case of acute obstructive pyelonephritis in a young man with genetically confirmed FAP. The pyelonephritis was directly attributable to ureteral compression by a previously diagnosed intra‐abdominal desmoid tumor. This case underscores the critical need for heightened clinical suspicion of desmoid‐induced urological complications in FAP patients presenting with flank pain or urinary infection symptoms. It emphasizes the essential roles of prompt, high‐resolution imaging and coordinated multidisciplinary care in preventing irreversible renal damage.

## 2 Case Presentation and Examination

A 36‐year‐old Iranian man with genetically confirmed familial adenomatous polyposis (FAP) presented to the emergency department with a 24‐h history of acute, deep, non‐radiating right flank pain that was aggravated by movement. Although the diagnosis of FAP had been established through genetic testing, the specific *APC* pathogenic variant was unavailable in the patient's medical records. His symptoms were accompanied by high‐grade fever (39.5°C), rigors, nausea, two episodes of non‐bilious, non‐bloody vomiting, and newly developed oliguria. He denied dysuria, urinary frequency, urgency, hematuria, or other lower urinary tract symptoms.

His medical history was significant for prophylactic total colectomy with end ileostomy performed in early 2022 following the diagnosis of FAP, which had been established in late 2021 based on colonoscopic identification of more than 1000 colorectal adenomas, confirmation of an *APC* gene mutation, and a strong family history of colorectal cancer. His father had been diagnosed with colorectal adenocarcinoma in 2020 and was undergoing oncologic treatment, while his paternal grandfather had died from colorectal cancer.

Surveillance imaging performed in early 2024 demonstrated a 14 × 10 × 5 cm intra‐abdominal mass involving the right lower quadrant and pelvis. Ultrasound‐guided core‐needle biopsy confirmed desmoid‐type fibromatosis. The patient subsequently received oncologic follow‐up and underwent multiple cycles of systemic chemotherapy.

On admission to the infectious diseases ward, the patient appeared acutely ill, diaphoretic, and volume‐depleted but was alert and oriented. Vital signs revealed fever (39.5°C), tachycardia (HR 105 bpm), hypotension (BP 90/54 mmHg), and normal respiratory parameters (RR 18/min, SpO_2_ 95% RA). The physical examination was significant for dry mucous membranes and marked tenderness to percussion in the right costovertebral angle. Abdominal examination revealed a soft, non‐distended abdomen without guarding or rebound; the ileostomy was viable with slightly increased output. Cardiopulmonary examination was notable only for sinus tachycardia. Review of systems was positive for significant fatigue, night sweats, unintentional weight loss (12% over 6 months), orthostatic lightheadedness, decreased appetite, and dyspepsia. There was no prior history of urinary tract symptoms or infections.

## 3 Differential Diagnosis, Investigations and Treatment

Initial laboratory studies revealed marked leukocytosis at 23,000/mm^3^, with neutrophil predominance (90%). Renal function tests demonstrated evidence of acute kidney injury, with serum creatinine elevated to 1.5 mg/dL (baseline: 0.8 mg/dL) and a concurrent rise in blood urea nitrogen (BUN). Urinalysis showed a positive leukocyte esterase test with significant pyuria (> 50 WBCs/high‐power field), but no hematuria or bacteriuria was detected.

Initial sonographic assessment revealed moderate right‐sided hydronephrosis, without evidence of nephrolithiasis or bladder distension.

A non‐contrast abdominopelvic CT scan was performed due to impaired renal function, precluding the use of intravenous contrast. The scan demonstrated postsurgical changes consistent with a total colectomy and right lower quadrant stoma placement.

**FIGURE 1:**
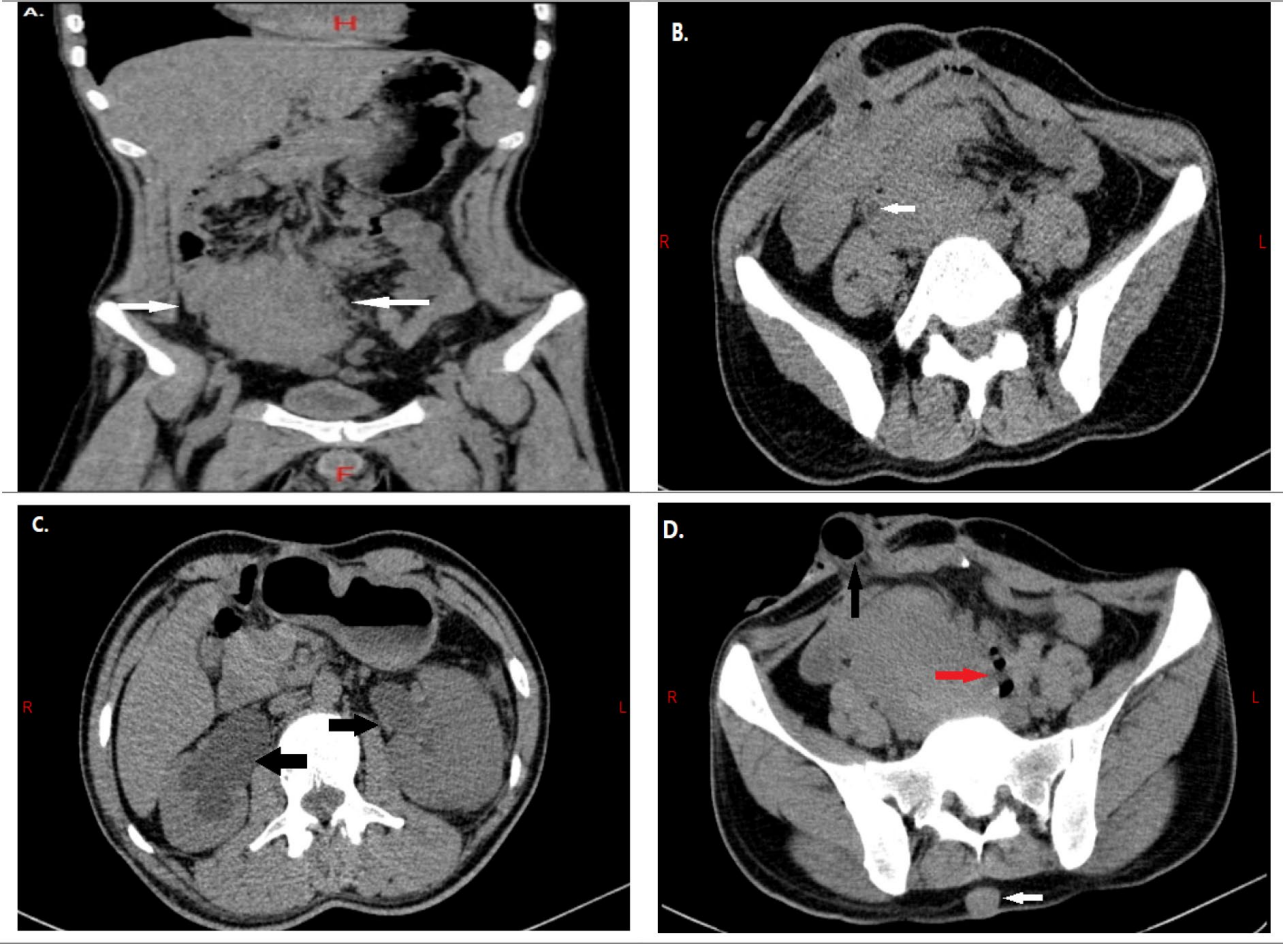
(A) Non‐contrast coronal abdominopelvic CT image demonstrating a large, infiltrative mesenteric mass (between white arrows) in the lower abdomen and pelvis, consistent with a desmoid tumor. The mass infiltrates the greater omentum, displaces small bowel loops, invades the right ureter, and compresses the left ureter, leading to moderate bilateral hydroureteronephrosis. (B) Non‐contrast axial abdominopelvic CT image, the mass is seen encasing and invading the right ureter (white arrow). (C) Non‐contrast axial abdominopelvic CT image demonstrating bilateral hydroureteronephrosis (black arrows), with visible dilation of both renal pelvicalyceal systems and proximal ureters. The findings are consistent with obstructive uropathy, attributed to an infiltrative mesenteric desmoid tumor encasing and compressing the ureters. (D) Non‐contrast axial abdominopelvic CT image showing post‐surgical changes consistent with prior total colectomy, including absence of colonic structures and evidence of stoma placement (black arrow) in the right abdominal wall. A large, ill‐defined desmoid tumor measuring approximately 11 × 7 × 12 cm is seen occupying the lower abdomen and pelvic cavity, with infiltration and displacement of adjacent small bowel loops (red arrow) and involvement of the right ureter, contributing to obstructive pyelonephritis. Additionally, a well‐defined, subcutaneous, 1 × 1 × 1 cm soft tissue lesion is noted in the posterior midline over the sacral region (white arrow), suggestive of a subcutaneous epidermoid cyst.

A large, ill‐defined, infiltrative soft‐tissue mass measuring approximately 11 × 7 × 12 cm was identified in the lower abdomen and pelvic cavity, likely of mesenteric origin, and was consistent with a desmoid‐type fibromatosis (Figure 1A). The mass displaces adjacent small bowel loops, compresses the right ureter (Figure 1B), and compresses the left ureter, resulting in moderate bilateral hydronephrosis (Figure 1C). No intratumoral calcifications or necrosis were identified. A well‐circumscribed, 1 × 1 × 1 cm soft tissue mass is noted in the subcutaneous tissue overlying the upper sacrum, consistent with an epidermoid (epidermal inclusion) cyst. This lesion appears homogeneous and sharply marginated, without calcification or invasion of deeper structures, supporting a benign etiology. Its presence may be incidental, but it could be related to the underlying diagnosis of FAP, which is known to include epidermoid cysts as an extracolonic manifestation (Figure 1D).

**FIGURE 2:**
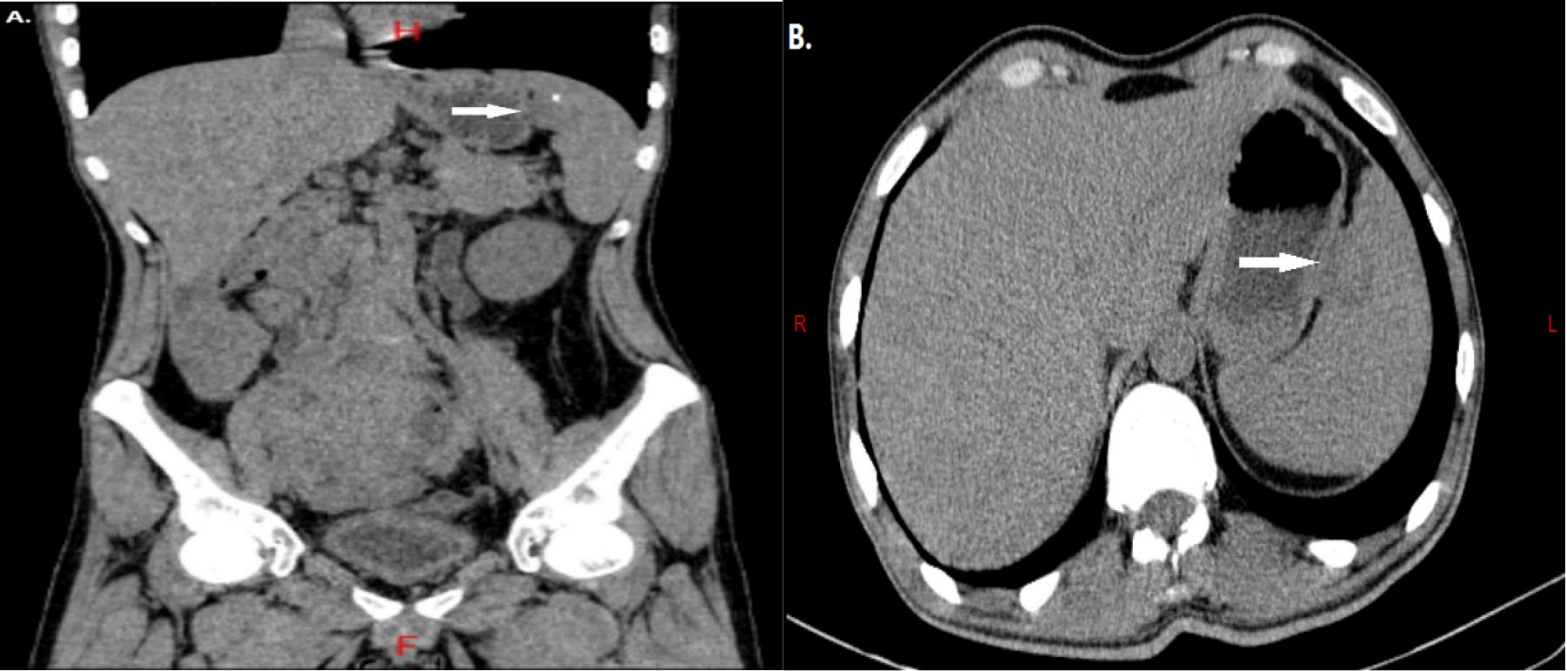
Non‐contrast (A) Coronal and (B) Axial views of a solitary, well‐circumscribed, hypodense, micro‐calcified, 2 × 2 × 2 cm lesion (white arrow) in the superior pole of the spleen, which is consistent with a splenic cyst, most likely a congenital epidermoid cyst, given the patient's history of familial adenomatous polyposis (FAP). No internal septations or solid components are observed.

A well‐defined, hypodense, subcapsular, 2 × 2 × 2 cm lesion was seen in the superior pole of the spleen, consistent with a benign simple cyst. In the context of FAP, an epidermoid cyst may be a potential etiology (Figure 2).

Comparison with the patient's prior surveillance CT scan performed in 2024 confirmed the persistence of the desmoid tumor but demonstrated interval progression, specifically with increased extrinsic compression of the right ureter and new compression of the distal left ureter. These findings directly correlated with the bilateral hydronephrosis observed on ultrasonography and CT scan. Importantly, no evidence of obstructing calculi or additional mass lesions was identified.

Based on the constellation of fever, flank pain, costovertebral angle tenderness, leukocytosis, pyuria, AKI, and confirmed hydronephrosis with a known compressing desmoid tumor, the diagnosis was obstructive pyelonephritis complicated by urosepsis (SOFA score: 2—Respiratory 0, Coagulation 0, Liver 0, Cardiovascular 1 [MAP < 70], CNS 0, Renal 1 [Creatinine 1.5]). The desmoid tumor was identified as the cause of ureteral obstruction.

Upon identification of ureteral obstruction from the desmoid tumor as the source of infection, immediate management was directed toward hemodynamic stabilization and source control. Sepsis resuscitation was initiated with aggressive intravenous crystalloid administration, totaling 3 L within the first 6 h, to address hypotension and volume depletion. This resulted in a gradual improvement of mean arterial pressure (MAP).

Empiric antimicrobial therapy was initiated with intravenous ceftriaxone 2 g daily, selected for its broad coverage against gram‐negative bacteria and favorable pharmacokinetic profile in urinary tract infections. Antimicrobial treatment was guided by local resistance patterns while awaiting definitive culture data.

Within 48 h, clinical improvement was evident: the patient's fever subsided, flank pain decreased, urine output increased, and leukocytosis began to resolve. Urine culture subsequently yielded *Escherichia coli* sensitive to ceftriaxone, confirming the appropriateness of the empiric therapy. Antimicrobial treatment was therefore continued for a full 10‐day course. Supportive measures, including careful monitoring of fluid balance, renal function, and hemodynamic status, were maintained throughout hospitalization.

## 4 Conclusion and Results

The patient's clinical course demonstrated a favorable response to therapy. By 24 h, urine output had normalized (> 0.5 mL/kg/h), reflecting improved renal perfusion. Fever resolved within 36 h, and by day 3, serum creatinine decreased to baseline (0.8 mg/dL), indicating recovery of renal function. Inflammatory markers and leukocytosis progressively declined in parallel with the patient's symptomatic improvement.

Repeat renal ultrasound prior to discharge confirmed partial resolution of hydronephrosis, consistent with improved urinary drainage despite persistent ureteral obstruction from the desmoid tumor. At the time of discharge, the patient had completed the prescribed 10‐day course of intravenous ceftriaxone, was hemodynamically stable, afebrile, and pain‐free, with laboratory values within normal limits.

**FIGURE 3:**
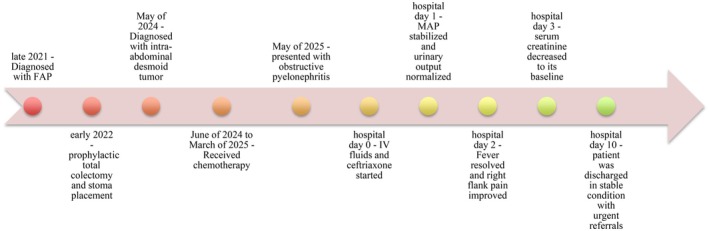
Timeline of events.

The patient was discharged with referrals to urology for consideration of definitive decompressive interventions such as ureteral stenting or percutaneous nephrostomy, and to oncology for comprehensive evaluation and management of the underlying desmoid tumor. The combined follow‐up plan emphasized both prevention of recurrent obstructive uropathy and initiation of disease‐directed therapy for the desmoid tumor. Figure 3 represents the timeline and chronological sequence of events of this case.

Desmoid tumors in FAP, while benign, can cause severe morbidity through ureteral obstruction and infection. Timely recognition, combined with cross‐sectional imaging and multidisciplinary management, is essential. Awareness of this rare but reversible complication is critical for preventing renal compromise and ensuring optimal patient outcomes in this high‐risk population.

## 5 Discussion

Desmoid tumors are a well‐recognized but challenging extracolonic manifestation of FAP, occurring in approximately 10%–15% of patients with FAP. Despite their histologically benign nature, their aggressive local infiltration and tendency to recur render them clinically significant sources of morbidity [2, 7]. Intra‐abdominal desmoids, particularly those arising in the mesentery or retroperitoneum, can compress adjacent organs, vessels, and ureters, resulting in severe complications such as bowel obstruction, ischemia, or hydronephrosis [8]. The present case illustrates an unusual but clinically important scenario in which a mesenteric desmoid tumor caused bilateral ureteral involvement and acute obstructive pyelonephritis.

Ureteral obstruction secondary to desmoid tumors is rare, with only scattered cases described in the literature [4, 5, 9]. Most commonly, desmoids cause chronic obstructive uropathy, whereas acute infection superimposed on obstruction, as observed in this patient, is exceptionally uncommon. The pathophysiology primarily involves progressive extrinsic compression of the ureters, resulting in impaired urinary drainage, hydronephrosis, and secondary infection. Importantly, nonspecific clinical manifestations such as flank pain, fever, or renal dysfunction may delay diagnosis unless a high index of suspicion is maintained in at‐risk individuals.

Cross‐sectional imaging is indispensable in identifying desmoid‐related ureteral obstruction. While ultrasound provides an initial non‐invasive assessment for hydronephrosis, CT scan and MRI offer superior anatomical delineation of tumor extent and involvement of adjacent structures [10, 11]. In this case, a non‐contrast CT scan was sufficient to establish the diagnosis due to pre‐existing renal impairment, highlighting its value in acutely unwell patients. Surveillance imaging is also recommended for patients with known intra‐abdominal desmoids, particularly those located near the ureters, as early recognition of obstruction can avert irreversible renal damage.

Management of desmoid tumors remains complex, as interventions themselves may precipitate tumor progression [12, 13]. Historically, surgical resection was considered first‐line [14]; however, high recurrence rates and the risk of exacerbating tumor growth have shifted practice toward conservative and medical approaches [12, 13]. Systemic therapies, including nonsteroidal anti‐inflammatory drugs, anti‐estrogen agents (e.g., tamoxifen), and tyrosine kinase inhibitors (e.g., sorafenib), represent mainstays of treatment, with radiotherapy or cytotoxic chemotherapy reserved for refractory cases [12, 13, 15]. In the context of ureteral obstruction, urological interventions such as ureteral stenting or percutaneous nephrostomy may be necessary to preserve renal function, but these should be carefully balanced against the risks of repeated procedures [5].

In the present case, prompt initiation of antimicrobial therapy and hemodynamic stabilization led to rapid resolution of the infection, and a multidisciplinary follow‐up plan was implemented to address the underlying obstruction. This reflects best practice, whereby acute infections are managed emergently, while longer‐term strategies focus on disease modification and renal preservation.

Optimal care for patients with FAP and desmoid tumors requires close collaboration between gastroenterologists, oncologists, surgeons, radiologists, and urologists. Surveillance protocols, patient education regarding symptoms of obstructive uropathy, and timely referral to urology are essential components of care. Tailored management should balance the goals of tumor control, renal protection, and quality of life.

This case adds to the limited body of literature describing desmoid‐induced obstructive pyelonephritis. It underscores the importance of considering desmoid tumors as a potential etiology for urinary obstruction and infection in patients with FAP, even in the absence of prior urinary symptoms. Additionally, the incidental finding of splenic and epidermoid cysts is consistent with the phenotypic spectrum of FAP, underscoring the need for comprehensive systemic evaluation in these patients.

## Author Contributions

**Alireza Mehrban:** conceptualization, data curation, formal analysis, funding acquisition, investigation, methodology, project administration, resources, software, supervision, validation, visualization, writing – original draft, writing – review and editing. **Mehdi Karimi:** conceptualization, data curation, formal analysis, funding acquisition, investigation, methodology, project administration, resources, software, supervision, validation, visualization, writing – original draft, writing – review and editing. **Mohammad Taghi Hamidian:** conceptualization, data curation, formal analysis, funding acquisition, investigation, methodology, project administration, resources, software, supervision, validation, visualization, writing – original draft. **Mahdi Masoumi:** data curation, formal analysis, funding acquisition, investigation, methodology, project administration, resources, software, supervision, validation, visualization. **Elahe Tari Najarkolaei:** data curation, formal analysis, funding acquisition, investigation, methodology, project administration, resources, software, supervision, validation, visualization, writing – original draft. **Abdolreza Babamahmoodi:** conceptualization, data curation, formal analysis, funding acquisition, investigation, methodology, project administration, resources, software, supervision, validation, visualization, writing – original draft, writing – review and editing.

## Funding

The authors have nothing to report.

## Ethics Statement

This case report was conducted in accordance with the Declaration of Helsinki. Ethical approval was obtained from the *Ethics Committee of Babol University of Medical Sciences*, Babol, Iran.

## Consent

Written informed consent was obtained from the patient for publication of this case report and any accompanying images. A copy of the written consent is available for review by the Editor‐in‐Chief of this journal.

## Conflicts of Interest

The authors declare no conflicts of interest.

## Data Availability

All data related to the case are reported in the manuscript. Contact the corresponding author if you require any further information.
